# Development of a Web-Based Mini-Driving Scene Screening Test (MDSST) for Clinical Practice in Driving Rehabilitation

**DOI:** 10.3390/ijerph19063582

**Published:** 2022-03-17

**Authors:** Myoung-Ok Park

**Affiliations:** Department of Occupational Therapy, Division of Health Science, Baekseok University, Cheonan-si 330-704, Korea; parkmo@bu.ac.kr; Tel.: +82-10-9999-8636; Fax: +82-41-550-2185

**Keywords:** driving rehabilitation, driving scene, screening test, web-based evaluation

## Abstract

(1) Background: For the elderly and disabled, self-driving is very important for social participation. An understanding of changing driving conditions is essential in order to drive safely. This study aimed to develop a web-based Korean Mini-Driving Scene Screening Test (MDSST) and to verify its reliability and validity for clinical application. (2) Methods: We developed a web-based MDSST, and its content validity was verified by an expert group. The tests were conducted with 102 elderly drivers to verify the internal consistency and reliability of items, and the validity of convergence with the existing Korean-Safe Driving Behavior Measure (K-SDBM) and the Korean-Adelaide Driving Self-Efficacy Scale (K-ADSES) driving tests was also verified. The test–retest reliability was verified using 54 individuals who participated in the initial test. (3) Results: The average content validity index of MDSST was 0.90, and the average internal consistency of all items was 0.822, indicating high content validity and internal consistency. The exploratory factor analysis for construct validity, the KOM value of the data, was 0.658, and Bartlett’s sphericity test also showed a strongly significant result. The four factors were road traffic and signal perception, situation understanding, risk factor recognition, and situation prediction. The explanatory power was reliable at 61.27%. For the convergence validation, MDSST and K-SDBM showed r = 0.435 and K-ADSES showed r = 0.346, showing a moderate correlation. In the evaluation–reevaluation reliability verification, the reliability increased to r = 0.952. (4) Conclusions: The web-based MDSST test developed in this study is a useful tool for detecting and understanding real-world driving situations faced by elderly drivers. It is hoped that the MDSST test can be applied more widely as a driving ability test that can be used in the clinical field of driving rehabilitation.

## 1. Introduction

The act of driving promotes community mobility and allows greater participation in community activities for the elderly and people with disabilities [1,2]. However, driving requires rapid decision-making and high-level cognitive processing to adapt to continuously changing situations and environments [2]. Compared with other tasks, driving requires continuous concentration and instantaneous concentration changes in a dynamically changing environment [3]. Declining cognition and perception caused by aging or damage to the central nervous system is one of the constraints that sometimes makes it difficult for some individuals to undertake meaningful work in daily life [4]. Symptoms such as deterioration of cognition and perceptual processing skills, visual deterioration, and slow physical reaction caused by aging deteriorate the quality of occupational performance in daily life [5]. Regarding driving performance, these physiological and psychological changes lead to a decrease in qualitative driving performance [6]. Among those factors, problems appearing as cognitive and perceptual impairments make it difficult to interpret the driving situation and may impair quick and accurate decision-making [7]. How well an individual reacts to a driving situation cannot be measured purely by simple cognitive and perceptual assessments. Many previous studies have reported that various evaluative tests can be used to predict driving performance skills [8,9] such as the visual function test to evaluate the visual skills required for driving, the Rey–Osterrith Complex Figure (ROCF), the Motor-Free Visual Perception Test-3 (MVPT-3), Trail Making Test-A (TMT-A), and Trail Making Test-B (TMT-B) [10,11,12]. However, these tests are only tools for examining basic cognitive and perceptual skills for driving performance, rather than evaluations of real-world driving situations.

According to a study by Fisk et al. [13], approximately 87% of stroke survivors who subsequently stopped driving did not have their driving skills assessed. This reflects the importance of an appropriate driving ability evaluation. In a review study targeting stroke patients, various factors related to driving were analyzed. Cognitive and perceptual factors were found to be the most influential factors affecting the driving performance of people who have suffered a stroke or brain damage [14]. In addition to nervous system damage, disorientation, loss of working memory, reduced cognitive processing speed and attention deficiency caused by aging can all adversely affect driving performance [15]. Therefore, elderly drivers must be able to evaluate their understanding of real-world driving situations.

Many evaluations based on real-world driving scenarios are evaluated by driving simulators [16,17]. This is because it is possible to predict whether a risk that may appear during on-road driving can be accurately perceived by the driver [18]. An example is a case of implementing and evaluating an off-road driving situation using a driving simulator [19]. However, in such cases, the simulator must be equipped with an evaluation setting meaning that it is difficult to perform accurate driving assessments in institutions that do not have expensive driving simulators. In addition, the driving scenarios in driving simulators are rarely standardized across all the different types of simulator. The driving simulator has the advantage that it can be expected to improve functions in various areas due to driving training in a simulated situation. However, compared to the lower age group, the older age group was shown to be at risk of dropping out due to symptoms such as dizziness, motion sickness, headache, fatigue, and nausea [20,21]. Studies have reported that it is difficult to directly apply actual driving performance assessments for stroke victims, people with brain damage, and for elderly people who are experiencing visual, perceptual, and cognitive deterioration because of cognitive and visual risk factors for physical and mental decline [22,23]. Therefore, a test that can be easily used in rehabilitation centers and does not just evaluate cognitive and perceptual factors is needed, along with assessment and training programs based on real-world driving situations.

In previous studies, some driving scene-based driving evaluations have been suggested, such as the NAB (Neurological Assessment Battery)-driving scene and the visual recognition slide test, which have been successfully applied after validation [24,25]. According to a recently published study, it was reported that the risk perception level was higher when examining driving performance on a driving simulation after computer-based driving recognition training for elderly drivers [26]. However, a web-based driving scene screening test that can be easily used by elderly drivers in Korea does not currently exist. Web-based tests have the advantage that users can easily access them and can immediately feedback their results. These tests also prevent the driving sickness that can occur during simulator tests or training [27].

Therefore, the purpose of this study is to develop a web-based simple driving scene screening test that can be easily applied in driving rehabilitation and to verify the reliability and validity of the test.

## 2. Materials and Methods

### 2.1. Study Design

This study was conducted from August 2019 to December 2020. In this study, the Delphi validity was verified by an expert focus group on the web-based driving scenario screening test, and the developed MDSST was tested by elderly drivers living in the local community in order to verify its validity and reliability. Ethical approval for the study was given by the IRB of Baekseok University (BUIRB-201907-HR-010). All participants read a description of the study, which described the purpose, methodology, procedure, and ethical issues based on the Declaration of Helsinki before providing written consent.

### 2.2. Research Procedures

This study consisted of three stages: (1) development stage of paper-based driving scene components; (2) conversion and final design of web-based driving scene components; (3) expert validation and verification.

Step 1: Steps in paper-based item configuration

Questions based on real-world driving scenarios were devised after examining relevant national and international literature and based on an understanding of domestic road driving laws. The levels of questions to be included in the driving scene were divided into stages, based on previous studies [28,29] and on the perception stage, i.e., asking for information about the basic level for understanding of the driving situation, the stage of understanding the driving scenarios, and the prediction stage for the driving scenarios [28].

A total of 30 questions, including steps, were formulated, that focused on standard driving laws, driving errors, and predicting dangerous situations. After a focus group discussion, with two doctoral-degree researchers with experience in conducting research on driving rehabilitation for the content of the first stage, two professors experienced in conducting driving rehabilitation research, and one clinical driving rehabilitation expert, a final total of 16 questions was agreed upon.

Step 2: Expert content validation and web-based MDSST configuration

When developing the web-based MDSST, the qualitative validity of the preliminary questions was verified via a focus group consisting of two web developers and three professors who had experience in driving rehabilitation research among the focus groups in the first stage (Appendix A final question). Verification depends on whether the question and the content of the item are consistent, whether the item accurately reflects a real-world driving situation, whether it contains a driving error, and whether the item corresponds with the perception, understanding, and prediction stages. Next, the CVI values were calculated. By using Fehring’s [30] method, the CVI was calculated on a five-point scale with weights of 0 for 1, 0.25 for 2, 0.50 for 3, 0.75 for 4, and 1 for 5. The weights of the scores assigned by the five experts for each task were then averaged. Implementing the web-based scenario consisted of a database for designing the hardware and software environments, user registration and login, evaluation guidance, evaluation execution, and result feedback. The program was designed so that the results page was immediately visible and the subject could provide self-feedback, while the result value was implemented so that only the examiner could see the statistics. All questions were answered within one minute, in consideration of immediate situation judgment, which requires quick understanding and response to the driving situation [31].

Constructing the web-based MDSST was carried out in cooperation with UnKim Soft, and the webserver was linked to the domain in Cafe 24 and presented.

Step 3: Real subject experiment-validation of web-based MDSST

This experiment was conducted to verify the validity of the web-based MDSST. The recruitment and testing phase was conducted from June 2019 to March 2020. All of the study participants, which consisted of 102 elderly people, had the ethics of the research process explained to them, and all participants consented to the study. The MDSST test link was then shared with the participants, and a voluntary evaluation was conducted.

The selection criteria for the study participants was that they had to be aged 60 or older with driving experience, and that they could read and understand the questions. To verify the validity, correlations were made with K-ADSES and K-SDBM, which are tools for measuring driving performance and safe driving behavior.

### 2.3. Assessments Used to Validate the Convergent Validity of the Web-Based MDSST

Korean-Safe Driving Behavior Measure (K-SDBM)

The K-SDBM is a self-reporting assessment of how older drivers comply with safety regulations in various driving scenarios. It consists of 37 questions (modified from the original 54 to suit domestic conditions) which were developed in 2014 and translated into Korean models.

Respondents answered questions related to driving, such as “driving in fog,” “driving at night,” and “driving on a narrow road” at four levels of difficulty (“very difficult (1),” “somewhat difficult (2),” “slightly difficult (3),” and “not difficult (4)”) based on their experiences over the last three months. The K-SDBM is an evaluation with proven reliability and a high internal consistency of 0.97 [32].

Korean-Adelaide Driving Self-Efficacy Scale (K-ADSES)

K-ADSES is a Korean-style self-driving efficacy scale developed by Park and Kim [32]. It consists of 12 topics that drivers normally encounter on a daily basis such as “driving in your local area” and “driving in heavy traffic.” The response for each item is based on a 10-point scale, ranging from 0 (not confident) to 10 (completely confident). According to a study by George et al. [33], the internal consistency of this assessment was 0.98, indicating high reliability. K-ADSES’s Cronbach’s α value for the entire item was 0.975. The test–retest reliability of K-ADSES indicated a significant correlation, with an Internal Classification Coefficient (ICC) of 0.813 [34].

### 2.4. Statistical Analysis

All data analysis was performed using SPSS version 20.0 (Armonk, NY: IBM Corp). For the general characteristics of the participants, we used descriptive statistics of mean, frequency, and percentage. We calculated the CVI index for the content validity analysis of MDSST and performed an exploratory factor analysis on a total of 15 questions to verify the validity of the MDSST composition. To verify the convergence validity of MDSST, Pearson’s correlation analysis was performed to determine any correlations between K-SDBM, a self-reported driving evaluation developed for Korean drivers, and K-ADSES, an evaluation of driving efficacy.

For reliability analysis, Cronbach’s α value was used for the internal consistency test, and Pearson’s correlation coefficient was used for the reliability analysis of the retest, which took place two weeks after the initial test. All statistical significance levels were selected as α = 0.05.

## 3. Results

### 3.1. General Characteristics of Participants

The average age of the participants was 67.33 ± 5.54 years, with 30.36 ± 11.92 years of driving experience. The participants consisted of 83 males (81.4%) and 19 females (18.6%). Regarding the type of driving license, 65.7% of the participants had level 1 licenses and 34.4% had level 2 licenses. Regarding driving habits, 81% stated that they drive 1–2 times a week, 12.7% drive 3–5 times a week, and 7.8% drive every day (Table 1).

### 3.2. Content Validity and Reliability of MDSST Each Item

Regarding the content validity analysis by the expert group for the MDSST, the average CVI score of all 16 items was 0.90, ranging between 0.82 and 0.96. Regarding the reliability analysis of each item, the average Cronbach’s α score was 0.822, and all items showed a distribution of more than 7 points, indicating a high degree of internal agreement (Table 2, Appendix A).

### 3.3. Construct Validity by Factor Analysis

In order to verify the validity of the configuration of the web-based MDSST, a factor analysis was performed using the results of the initial evaluation of the participants. After examining the factor fit of the data, Bartlett’s sphericity test statistic (df 120, *p* < 0.001, chi-squared: 709.193) showed satisfactory results.

The Kaiser-Meyer-Olkin (KMO) value of all the results was 0.658, indicating that the model was suitable. After analyzing the factor structure through Berry’s pseudo-rotation, four principal component analyzes were grouped, factors 1, 2, 3, 4, 5, 6, 11 questions, 7, 9, 15 questions, and Factor 3 Questions 8, 14, and 16 were included in Factor 4, and questions 10 and 13 were included in Factor 4. The cumulative variance for the total variance of the factors was 61.27%. As for the extracted sub-factors, Factor 1 of the MDSST was road traffic and signal perception, Factor 2 was situation understanding, Factor 3 was risk factor recognition, and Factor 4 was situation prediction (Table 3).

### 3.4. Convergent Validity of Web-Based MDSST

To verify the convergence validity of the web-based MDSST, we analyzed its correlation with both K-SDBM and K-ADSES. The total score of K-SDBM and MDSST was r = 0.435 (*p* = 0.000), and the total score of K-ADSES and MDSST was r = 0.0346 (*p* = 0.000), indicating a significant correlation. When examining the relationship with the sub-items, the K-SDBM total score and items 1–7, 9, 11, and 16 among the MDSST sub-items showed a significant correlation. The K-ADSES total score and items 1, 3–9, and 11 of the MDSST sub-items also showed a significant correlation (Table 4).

### 3.5. Test–Retest Reliability of Web-Based MDSST

**Figure 1:**
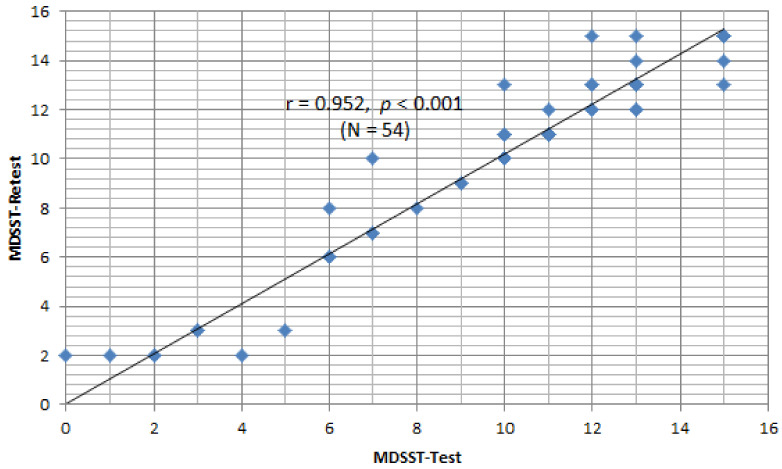
Test–retest reliability of web based-MDSST.

The retest for the web-based MDSST was performed two weeks later with 54 of the participants from the initial evaluation. The Pearson correlation analysis showed that the reliability between the initial evaluation and the reevaluation was r = 0.952 with *p* = 0.000, indicating a very reliable linear correlation (Figure 1).

## 4. Discussion

In order to drive safely, motorists need visual, auditory, cognitive, perceptual, and motor skills and must also be able to interact with their surrounding environment [35]. However, for elderly drivers, physical, cognitive, and perceptual changes due to aging can make driving difficult. Hu et al. [36] reported that physical, sensory, and cognitive changes that occur with age can affect driving performance. Tests of driving performance include tests of cognitive and perceptual functioning, along with driving suitability tests to determine whether safe driving is possible [37]. In this study, we developed a web-based test to determine whether drivers are aware of road traffic regulations and driving conditions. Most of the tests on existing driving conditions were based on those found in driving simulators. However, driving simulators are expensive to install and operate, particularly in rehabilitation centers. Therefore, our web-based MDSST was developed in order to be a simple and brief evaluation of a user’s driving skills with the results immediately available to the user so that they can self-evaluate. It also has the advantage of being able to easily measure whether a task is difficult or not.

Looking at the research results, based on the content validity verification of the MDSST by the expert focus group, the CVI score of all 16 items showed an average of 0.90 points with a range of 0.82–0.96, indicating a high content validity. The CVI score was judged as a meaningful score, based on a score of 0.75 for each item based on previous studies [30]. All 16 items had a CVI score of 0.75 or higher, indicating that all the items adequately reflected real-life driving situations for each question.

The internal consistency of the items in the MDSST was verified. The average Cronbach’s α value was 0.822, and all items showed a distribution of more than 7, indicating a high degree of internal agreement. Among the sub-items, the item with the lowest Cronbach’s α value was Item 4 (are you currently driving on a highway? Is it a national road?), which had a score of α = 0.796, while the item with the highest Cronbach’s α value was Item 15 (what factors should be considered when turning right in the current driving situation?), with a score of α = 0.824. The results indicated that the MDSST is a reliable tool.

Regarding the construct validity test to see whether the items converge to factors that can evaluate the perception of the driving situation through exploratory factor analysis, the KOM value of the data was 0.658, and Bartlett’s sphericity test showed a strongly statistically significant result.

Each item was classified into four factors. The items included in Factor 1 were generally related to the level of perception for recognizing basic driving conditions such as signals, sign meanings, recognition of visible lanes, and weather conditions. Factor 2 included questions about situational awareness, and items corresponding to the number of pedestrians in the current driving situation, recognizing the farthest vehicle, and recognition of situations to be considered when turning over. Factor 3 included items for perceiving risk factors in more complex driving situations, while Factor 4 included items for predicting potentially dangerous situations. When making decisions in dynamic situations, such as driving a vehicle, situational awareness and accurate decision-making skills are essential. According to a previous study, each stage is perception/recognizing of important elements in the state and characteristics of situation-related elements, and refers to the state of recognizing without additional information processing [38].

The comprehension stage is where the perceived information is interpreted and understood according to the operational purpose. In the projection stage, future situations are predicted based on the information from the perception and understanding stage, and the perception of safety is obtained by self-feedback through appropriate decision-making [28,39]. As a result of the exploratory factor analysis, it was found that the factors based on the situational awareness model were well reflected in the dynamic driving situation scene in our MDSST. The cumulative variance of the total variance of the factors was 61.27%, indicating a relatively stable explanatory power.

We also verified the validity of convergence between the MDSST and self-driving evaluation for elderly drivers applied in clinical practice. For convergent validity, we analyzed correlations between K-SDBM and K-ADSES. As a result of the verification, the total score of the K-SDBM and the total score of the MDSST showed a moderately significant correlation, with a correlation coefficient of 0.435. In addition, the K-ADSES total score and the MDSST total score, showed a weak but significant correlation, with a correlation coefficient of 0.346. This means that our MDSST is useful for determining the level of recognition of driving functions for elderly drivers. Previous studies have reported that the higher the safe driving behavior score, the higher the driving performance score [32].

Regarding novice drivers, it was reported that errors increased and feelings of driving efficacy decreased because of their limited understanding of driving concepts such as awareness and the ability to cope with changing situations on the road [40]. Understanding the current driving situation on the road is therefore fundamental for safe driving.

In addition, since the web-based MDSST developed in this study is a test that simulates actual scenarios rather than a simple questionnaire of driving suitability test, it is thought that it will be able to more accurately and clearly measure a subject’s understanding of the driving situation. Another previous study reported that, when feelings of driving efficacy were low, the frequency of driving mistakes and traffic law violations increased [41,42]. This means that self-efficacy for driving also affects driving performance on the road. Future studies should conduct actual road driving tests in combination with the MDSST to determine how much the MDSST score is related to real-world driving on the road and to determine whether driving efficacy and driving situations are understood when actual driving tests cannot be performed. It is necessary to verify predictability.

The retest for the web-based MDSST was performed two weeks later with 54 participants who had also participated in the initial evaluation. The Pearson correlation analysis showed that the reliability between the initial evaluation and the reevaluation was very high, with a correlation coefficient of 0.952. This means that the evaluation-reevaluation reliability of the MDSST developed in this study shows very consistent reliability. A previous study by Brown et al. [43] noted that there was a degree of correlation between the driving scene test and actual driving performance when conducting a neuropsychological evaluation of elderly people with very mild dementia. The examination was performed by asking the participants to recognize illustrations of driving situations presented as paper-type pictures rather than a web-based examination [43]. It differs in that it reflects the actual driving situation well. The study only applied exploratory factor analysis during the validation process through factor analysis, although the model fit index will also need to be checked via confirmatory factor analysis.

In addition, the number of participants in the test–retest reliability verification process was significantly smaller than the number of initial examiners. Therefore, more participants will be necessary in order to conduct test–retest reliability studies via other groups in the future. A study will also be needed to determine how much it is related to the Mini-Mental Status Examination Test (MMSE) with MDSST, a test that is basically applied as a cognitive function evaluation scale for the elderly, and to present a cutoff score for driving performance through two evaluations.

Overall, however, and despite some limitations, this study is meaningful in that it developed a web-based MDSST that can be easily applied and utilized in the clinical field of driving rehabilitation.

## 5. Conclusions

The web-based MDSST developed in this study showed high validity as an evaluation reflecting driving conditions, and the test–retest reliability for the same group was also very high. The web-based MDSST is a useful driving site screening tool that can be simply applied in the driving rehabilitation clinical field.

## Figures and Tables

**Table 1:** General characteristics of participants (*n* = 102).

Variables	M ± SD, *n* (%)
Age (yr.)	67.33 ± 5.54
Driving experience (yr.)	30.36 ± 11.92
Gender	
Male	83 (81.4)
Female	19 (18.6)
Driver’s license type	
Level 1 ^(a)^	67 (65.7)
Level 2 ^(b)^	35 (34.3)
Driving habits (per week)	
Every day	8 (7.8)
1 to 2 times	81 (79.5)
3 to 5 times	13 (12.7)
Car type	
Sedan	82 (80.39)
Truck	10 (9.8)
Van	4 (3.92)
Other	6 (5.88)
Specific disease	
Yes	13 (12.7)
No	89 (87.3)

^(a)^ Level 1: General 15 people or less passenger cars. ^(b)^ Level 2: General 10 people or less passenger cars.

**Table 2:** Content validity and reliability of web-based MDSST (*n* = 102).

Sub-Items *	CVI	Cronbach’s α
Item 1	0.96	0.800
Item 2	0.92	0.800
Item 3	0.85	0.808
Item 4	0.92	0.796
Item 5	0.92	0.798
Item 6	0.92	0.809
Item 7	0.92	0.803
Item 8	0.92	0.828
Item 9	0.85	0.809
Item 10	0.96	0.823
Item 11	0.96	0.805
Item 12	0.92	0.813
Item 13	0.82	0.820
Item 14	0.85	0.820
Item 15	0.82	0.830
Item 16	0.82	0.824
Total Mean	0.90	0.822

* The sub-items are presented in Appendix A.

**Table 3:** Exploratory factor analysis for web-based MDSST (*n* = 102).

KMO Goodness-of-Fit (MSA) Test		0.658			
Bartlett’s Sphericity Test	Approx. x^2^	709.193			
	Degrees of freedom(df)	120			
	*p*	0.000			
*p* < 0.001					
Sub-Items	Factor 1	Factor 2	Factor 3	Factor 4	Communality
Item 1	0.840	0.006	0.048	0.036	0.710
Item 2	0.739	0.081	0.265	0.139	0.641
Item 3	0.559	0.389	−0.222	−0.034	0.514
Item 4	0.733	0.221	0.233	0.202	0.681
Item 5	0.834	0.189	−0.088	0.010	0.732
Item 6	0.643	−0.215	0.064	0.311	0.560
Item 7	0.471	0.637	0.326	−0.093	0.743
Item 8	0.145	0.383	−0.539	0.031	0.659
Item 9	0.323	0.524	−0.013	0.349	0.500
Item 10	0.071	0.302	−0.318	0.739	0.744
Item 11	0.706	0.057	0.128	0.028	0.519
Item 12	0.392	0.268	−0.170	0.267	0.526
Item 13	0.157	−0.087	0.263	0.777	0.705
Item 14	0.178	0.083	0.769	0.227	0.681
Item 15	−0.116	0.787	−0.016	0.041	0.635
Item 16	0.305	0.146	0.673	−0.290	0.651
Factor Name	Perception of Signs and Signals	Situation Comprehension	Risk Factor Awareness	Situation Prediction	
Eigenvalues	5.167	1.994	1.417	1.226	
Explanation Variance (%)	32.294	12.460	8.854	7.659	
Cumulative Variance (%)	32.294	44.754	53.608	61.267	

Extraction Method: CFA(common factor analysis). Rotation Method: Varimax.

**Table 4:** Correlations between MDSST, K-ADSES and K-SDBM (*n* = 102).

MDSST	K-SDBM ^a^	K-ADSES ^b^
Sub-Items	r	r
Item 1	0.597 **	0.480 **
Item 2	0.516 **	0.382
Item 3	0.371 **	0.409 **
Item 4	0.457 **	0.415 **
Item 5	0.539 **	0.486 **
Item 6	0.318 **	0.296 **
Item 7	0.410 **	0.283 **
Item 8	0.119	0.215 *
Item 9	0.277 **	0.211 *
Item 10	0.081	0.058
Item 11	0.343 **	0.331 **
Item 12	0.191	0.152
Item 13	0.144	0.056
Item 14	0.018	0.036
Item 15	−0.042	−0.113
Item 16	0.215 *	0.097
MDSST Total	0.435 **	0.346 **

* *p* < 0.05, ** *p* < 0.01, ^a^: total score of K-SDBM, ^b^: total score of K-ADSES, r: Pearson’s coefficient.

## Data Availability

The data is available from the corresponding author on reasonable request.

## Appendix A. Item Query List of Web-Based MDSST

**Items**	**Query**	**Scene Answer**
Item 1	What are the colors and meanings of traffic lights in the current driving situation?	Red, vehicle stop
Item 2	What does the sign mean in the current driving situation?	Driving under 60 km/h
Item 3	How many lanes are visible in the current driving situation?	Two lanes
Item 4	Are you currently driving on a highway? Is it a national road?	Highway
Item 5	What is the weather like in your current driving situation?	Raining
Item 6	What does the sign mean in the current driving situation?	Front speed bump
Item 7	How many pedestrians are there in the current driving situation?	Two
Item 8	What color is the farthest visible vehicle in your current driving situation?	Black
Item 9	What is the vehicle turning right in the current driving situation?	Van
Item 10	I want to go straight on in the current driving situation. In which lane should I drive?	Right side
Item 11	What should the driver do in the current driving situation?	Slow down
Item 12	What are the risk factors to consider in the current driving situation?	Front right side truck
Item 13	What are the risk factors to consider in the current driving situation? (Night)	Forward reverse motorcycle
Item 14	What are the risk factors to consider in the current driving situation?	Forward right side old man
Item 15	What factors should be considered when turning right in the current driving situation?	Left-side black car
Item 16	What are the risk factors to consider in the current driving situation? (Night)	Pedestrian in front
